# Sulphate of Cinchonine

**Published:** 1826-07

**Authors:** 


### ANALECTA MINORA,

OR

### *MINOR PERISCOPE.*

Nihil est aliud magnum quam multa minuta.

1. *Sulphate of Cinchonine.* It is known that the pale bark, or cinchona officinalis, yields a great deal of cinchonine and very little quinine.—The red bark, or cinchona oblongifolia, yields about equal parts of quinine and cinchonine—while the yellow bark, or cinch. cordif. yields a great deal of quinine and very little cinchonine.—Now, the immense demand which, of late years, has been made for quinine, has naturally produced both a scarcity and dearness of the yellow bark from which it is obtained, and this circumstance will infallibly lead to adulteration and deterioration of the medicine. It is, therefore, very desirable that trials should be made of the cinchonine, procurable from the other and less costly species of bark. M. Bailly has lately made a considerable number of experiments in the treatment of intermittent fevers, at La Pitié, on the febrifuge powers of the cinchonine and the results are favourable. The doses were generally from 12 to 16 grains of the medicine in the apyrectic interval, and the paroxysms were soon brought to an end. Although the cinchonine may be inferior to the quinine, yet it would be very desirable to introduce the former into practice on many accounts.

